# Transactions of the Gynecological Society of Chicago

**Published:** 1891-07

**Authors:** 


### TRANSACTIONS OF THE GYNECOLOGICAL SOCIETY OF CHICAGO.

---

### REGULAR MEETING, JANUARY 16, 1891.

DR. W. W. JAGGARD, President, in the chair.

DR. L. L. MCARTHUR read a paper on

CANCER OF THE RECTUM.*

DR. C. T. PARKES: The case as presented by Dr. McArthur is very interesting to me, and I think he is to be complimented on the skillful attention he has given to this patient and the success which has resulted from his interference. It seems to me we must look upon this operation for the relief of this terrible disease mainly as a palliative treatment; it is seldom curative. Certainly it removes the manifestations of the disease for a time, and, above all, is desirable, as Dr. McArthur has said, from the fact that it relieves the patient from the local distress caused by the disease, especially from pain, which is present in all these cases, and the symptoms of on-coming obstruction which accompany the later stages. My experience with it has been rather moderate; as I recall the cases I have met, and have thought a little about them since receiving the notice of this meeting, there come to mind *nine* cases in which operation has been done for excision of the rectum, two cases in which simple incision was done, and two cases which are interesting from the fact that they accompanied the presence of ovarian tumors, and one case which was

---

*See page 274.

situated very high in the rectum and no interference was practiced—in all fourteen. Of the nine cases in which excision was done, five were operated upon according to the plan of Kraske; in the others success was attained in the removal of the manifestations of the disease by merely external incision of the soft parts, without interference with the sacrum or coccyx.

Of these cases, which represent a period of work of eight or ten years, some are living to-day, but most of them are dead. None of the cases of excision were preceded by an opening into the colon. I think the statistics which the doctor gives as to the mortality of the disease, as the result of immediate excision, is based upon the results of pre-antiseptic days rather than the present. I am not one of those who believe that the contact of fecal matter with the wound is at all times hurtful, as I have had in my experience many cases in which wounds have been bathed in fecal matter without any septic condition following.

I can see readily enough that the previous operation for an artificial anus can be a benefit to these cases, and will likely predispose to the earlier and more rapid healing of the rectal wound, simply because it prevents the fecal material from passing over the raw surface. The operation of forming an artificial anus in itself is of little consequence, and should be, as a rule, attended with little fatality. That it is a necessary procedure I am not inclined to believe; neither do I think that it makes very much difference in the mortality. As far as the relief given in preventing the discharge from coming over the wound is concerned, I have to agree with Dr. McArthur's statements.

The disposition is, in all these cases, to a comparatively rapid return of the disease. We must always remember, in the treatment of cancer here, as well as elsewhere, that the operation itself may stimulate or produce infection. In two cases in which the operation was done by myself, there followed no local manifestations of return of the disease, but, within eighteen months their appeared to be general infection of the entire body, as shown by evidence of disease in the liver and in the lung, and the presence of cancerous nodules of different sizes in the integument.

There is no question but that every one of these patients will be grateful to the surgeon for the removal of the manifestations of this disease; but, as I said before, we must look at it in the true light and tell these patients that the relief is only temporary and cannot often be curative where the disease is really cancerous in its nature. Again we must bear in mind that quite a number of surgeons of great experience, men who have seen this disease in all its conditions and ravages—believe that the establishment of an artificial anus itself is a sufficient relief to the case. Dr. Thomas Bryant, of London, is not an advocate of excision, but is an advocate of an artificial anus and relieving the patient entirely of the necessity of using the diseased portion of the bowel for the transmission of fecal matter, and thereby allaying inflammation.

Again, we must remember that other operations are done besides excision of the rectum, which is a formidable operation and leaves disgusting results in many cases. Some other opertions have been done which surgeons of experience believe to be efficacious; these are local in character—that is, the complete division of the mass backward toward the sacrum, in that way providing for the easy exit of the fecal matter. Of course the era of operative procedure is upon us, and particularly is this operation advocated by European surgeons, and also very largely by American surgeons; but I think if all the cases were examined as carefully as those cases have been which Dr. McArthur has presented to us to-night, and discussed as coolly and calmly as he has discussed them, none of us would be very much in favor of promising a great deal for the operation.

DR. HENRY T. BYFORD: I would like to emphasize what Dr. Parkes has said, that this is a palliative operation and not justifiable when it is immediately very dangerous to the life of the patient. The case related was very interesting to me, because it is similar to a case which I have treated, and which illustrates the principle which should be carried out in treating cancer of the rectum in women. In this case the sphincter was not involved, although the rectum from about two and a half above down nearly to the sphincter was affected on its anterior and lateral aspect. I removed portions of the lateral and anterior rectal walls, and the posterior vaginal wall. Instead of drawing the parts together in front of the rectum, I operated upon the principle that all raw tissue not covered by mucous membrane will contract and obliterate the entire tract within it; so I endeavored to secure as large a surface of mucous membrane for the canal as possible by leaving the vagina open and merely closing up the vaginal entrance. There is another reason for removing, in cancer of the rectum, all of the rectum that we can, viz., that a return of the disease in connective tissue is not as painful as when it attacks the viscera. In this case the patient was able to evacuate the bowels until the entire pelvis was filled up with a mass of carcinomatous tissue, without very much pain. She died finally of exhaustion more than anything else. The point in all these cases is to get as much mucous membrane as possible, using the vagina the same as in any other operation.

DR. L. L. MCARTHUR, in closing the discussion, said: In presenting the patient this evening I did not do so to advocate such an operation, although it does seem to me that, in cases in the female in which carcinoma occurs low down in the rectum, in reality it would be a procedure more advisable than to make an artificial anus at one side of the tip of the coccyx, because of the statement of the patient that she knew when the bowels desired to move. She had a peculiar feeling, she says, and has the power to expel the contents, thus escaping the exceedingly distressing symptoms of involuntary discharges, which always occur with an artificial anus at other points. In regard to the statistics which I quoted as collected by Kelsey, in the article which he wrote on this subject for the "Reference Hand-book of Medical Sciences," published in 1886, he stated that the mortality from the operation of excision was thirty-three per cent. In Sajous' *Annual* for 1890, there is a collection of statistics after Kraske's operation, and the mortality is stated at fifty per cent. Kelsey happens also to be a contributor to this department of Sajous' *Annual*, so the mortality has rather increased than decreased for the last year, according to the same man's statistics. He bases this fifty per cent. mortality on seven cases done by Kraske himself, three by Schonborn, one by Rinne, and the remainder by Lauenstein, twelve in all, in which six died as the result of the operation ; some by septic peritonitis (two), some by sepsis (two), and some from exhaustion.

As Dr. Parkes says, there are a large number of surgeons who advocate simply making a colostomy and interfering to no further extent with the case. In some excellent statistics collected by Cripps, we find that out of cases which he watched personally in London hospitals and studied carefully, life was lengthened on the average from seventeen months to twenty-two months—that is, there were five and a half months added to the longevity by simply making a colostomy ; which shows that it is decidedly advantageous. Colostomy, I believe, will aid in lowering the mortality in cases of excision, whether done for pain or for obstruction. As to the operation being of dubious value, there is much to be said on both sides ; there are, however, some well-recorded cases in which, the operation having been performed at an early day, the life of the patient has been preserved. One case was living in 1886 that had been living for seven years since the operation, and no return was to be seen, and five cases in which no return was seen in two years.

It has impressed me that some of the gynecologists of the Society would comment upon the probability of endometritis with infection through the uterine canal and tubes as being very likely to occur in such a procedure, as was exhibited in my patient, and I would like to ask the President whether such an inflammation would be probable in a woman who had passed the menopause.

